# Design of a foundational sciences curriculum: Applying the ICAP framework to pharmacology education in integrated medical curricula

**DOI:** 10.1002/prp2.762

**Published:** 2021-05-11

**Authors:** Kelly M. Quesnelle, Naunihal T. Zaveri, Stephen D. Schneid, Joe B. Blumer, John L. Szarek, Marieke Kruidering, Michael W. Lee

**Affiliations:** ^1^ Department of Biomedical Sciences Western Michigan University Homer Stryker M.D. School of Medicine Kalamazoo MI USA; ^2^ Department of Integrated Medical Sciences College of Medicine Florida Atlantic University Boca Raton FL USA; ^3^ Skaggs School of Pharmacy and Pharmaceutical Sciences University of California San Diego La Jolla CA USA; ^4^ Department of Cell and Molecular Pharmacology and Experimental Therapeutics Medical University of South Carolina Charleston SC USA; ^5^ Department of Medical Education Geisinger Commonwealth School of Medicine Scranton PA USA; ^6^ Department of Pharmacology College of Medicine University of California San Francisco CA USA; ^7^ Department of Medical Education Department of Oncology Live Strong Cancer Institutes University of Texas at Austin Austin TX USA; ^8^ Present address: Arkansas College of Osteopathic Medicine Fort Smith AR USA

**Keywords:** active learning, basic science, ICAP framework, integration, medical education, multi institutional, pharmacology, pharmacology educator, PhIG, pre‐clinical

## Abstract

Expectations for physicians are rapidly changing, as is the environment in which they will practice. In response, preclerkship medical education curricula are adapting to meet these demands, often by reducing the time for foundational sciences. This descriptive study compares preclerkship pharmacology education curricular practices from seven allopathic medical schools across the United States. We compare factors and practices that affect how pharmacology is integrated into the undergraduate medical education curriculum, including teaching techniques, resources, time allocated to pharmacology teaching, and assessment strategies. We use data from seven medical schools in the United States, along with results from a literature survey, to inform the strengths and weaknesses of various approaches and to raise important questions that can guide future research regarding integration of foundational sciences in medical school and health professions’ curricula. In this comparative study, we found that there is significant heterogeneity in the number of hours dedicated to pharmacology in the preclerkship curriculum, whereas there was concordance in the use of active learning pedagogies for content delivery. Applying the ICAP (Interactive, Constructive, Active, Passive) Framework for cognitive engagement, our data showed that pharmacology was presented using more highly engaging pedagogies during sessions that are integrated with other foundational sciences. These findings can serve as a model that can be applied beyond pharmacology to other foundational sciences such as genetics, pathology, microbiology, biochemistry, etc.

## Graphical abstract

Comparison of pharmacology education practices at seven U.S. Allopathic medical schools revealed a mixture of teaching sessions where pharmacology was taught independently or in an integrated manner with other disciplines. Application of an active learning framework to the pedagogies used at these institutions suggests that integrated sessions are more active compared to standalone pharmacology sessions. We suggest a careful balance needs to be achieved between active and passive teaching approaches to avoid fragmenting and diluting the flow of topics in pharmacology education.

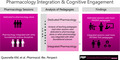

## 1 INTRODUCTION

Institutional emphasis on accreditation standards, coupled with evidence from educational and cognitive psychology literature, is driving dramatic changes to undergraduate medical education (UME) curricula across the United States and worldwide.^1^, ^2^ The last decade has seen shifts in medical education designed to prepare future physicians as life‐long learners who will deliver cost‐effective care in teams, using electronically available facts to improve the healthcare system.^3^ For years, disciplines such as pharmacology had their own discipline‐specific course in the UME curriculum. Now, however, basic science disciplines are integrated into organ‐system blocks of instruction as longitudinal threads within the preclerkship curriculum.^4^, ^5^, ^6^, ^7^, ^8^ This restructuring has occurred along with curricular changes aimed at providing earlier exposure to patient care, integration of health systems sciences, incorporation of more team‐based learning activities, adoption of competency‐based assessment practices and a greater emphasis on use of new technologies. While many courses are highly integrated, integration at the individual session level is variable, and there is a paucity of data available.

Medical education pedagogies are increasingly guided by findings from experimental studies on student learning from the medical education and psychology literature.^9^, ^10^, ^11^, ^12^ Didactic content delivery occurs in ways that use technology to enable asynchronous content delivery and allow the learner to control pace, timing, and learning sequence. Sessions are intentionally built around cognitive theories and, as such, many sessions include active learning strategies such as team‐based learning (TBL), problem‐based learning (PBL), case‐based learning (CBL), and simulations, which provide opportunities for students to apply what they have learned.^7^ The ICAP framework for cognitive engagement can be used to categorize educational pedagogies as Interactive, Constructive, Active, or Passive.^13^ In modern medical curricula, these pedagogies co‐exist, permitting a variety of individual learning styles among a singular student body.^14^, ^15^ Problematically, there is little guidance for faculty on which approaches are best suited for specific types of activities that have varying degrees of integration with other foundational and clinical sciences.

Despite many published examples of both integrated curricula and educational strategies, there is a dichotomy in the existing literature. Existing studies tend to either describe the entire curriculum broadly (AAMC Curriculum Survey, https://www.aamc.org/data‐reports/curriculum‐reports/report/curriculum‐reports), or they describe singular, narrowly focused interventions. This can make it difficult for content‐expert instructors, who are responsible for weaving content throughout the curriculum, to determine pedagogies suitable for the desired level of cognitive engagement at the session level. There is a paucity of data describing pharmacology content integration throughout the UME curriculum. Thus, when taken together, with the prominent role pharmacology knowledge plays in clinical clerkships, there is a clear need to understand how to optimally deliver pharmacology content to medical students that leads to durable long‐term retention and recall.^16^

Our multi‐institutional collaborative comprised faculty from seven allopathic medical schools in the United States and was aimed at identifying elements of curricular integration that contribute to effective pharmacology education within the preclerkship curriculum. In this descriptive study, we detail trends observed at our own institutions for pharmacology instruction in the preclerkship medical curriculum and contextualize those trends in the literature that currently exists for this type of collaborative work. We apply the ICAP framework to the most‐commonly utilized pharmacology pedagogies and compare the level of cognitive engagement required from students in dedicated pharmacology sessions versus integrated sessions that include pharmacology. This descriptive work will be useful for other pharmacology content experts as they engage in curriculum design and reform, and it may serve as a model for other foundational science content experts to develop similar collaboratives and studies.

## 2 MATERIALS AND METHODS

### 2.1 Literature survey for preclerkship pharmacology curricula

We conducted a PubMed search in 2020 with the assistance of the Medical Library at Dell Medical School at the University of Texas at Austin. We identified 326 papers using the following search strategy which incorporated keywords/phrases and MeSH terms:

(((("Education, Medical, Undergraduate"[Mesh]) OR "Schools, Medical"[Mesh]) OR "Students, Medical"[Mesh])) OR ("medical school"[Title/Abstract] OR "medical schools"[Title/Abstract] OR "medical student"[Title/Abstract] OR "medical students"[Title/Abstract])) AND (curriculum[Title/Abstract] OR curricula[Title/Abstract] OR curriculum[MeSH Terms])) AND ((pharmacology[MeSH Major Topic]) OR pharmacology[Title/Abstract]))) OR (((("Education, Medical, Undergraduate"[Mesh]) OR "Schools, Medical"[Mesh]) OR "Students, Medical"[Mesh])) OR ("medical school"[Title/Abstract] OR "medical schools"[Title/Abstract] OR "medical student"[Title/Abstract] OR "medical students"[Title/Abstract]))) AND "Pharmacology/education"[Mesh]) Filters: Publication date from 2000/01/01 to 2020/09/29; English.

From these 326 papers, we excluded descriptions of pharmacology instruction beyond the preclerkship year(s) and descriptions of instruction in non‐medical allied health professions’ programs. Based on this exclusion criteria, we reviewed 132 papers describing pharmacology education in the preclerkship curriculum of medical schools. We excluded studies describing singular learning events or course interventions and were left with 24 papers, that contained detailed descriptions of pharmacology curriculum in the preclerkship setting.

### 2.2 Comparative curriculum inventory

All authors reviewed the preclerkship foundational sciences curriculum at their respective institutions during academic year 2018–2019. General properties of the foundational sciences curriculum were captured, including length of preclerkship program, integration of curriculum, and timing of the national licensing exam USMLE (United States Medical Licensing Exam, administered by the National Board of Medical Examiners). In addition, pharmacology‐specific curricular elements were captured, including teaching hours and pedagogies, discipline‐specific assessment requirements, resources available (i.e., textbooks, commercially available programs), etc. We also collected information (hours and pedagogies) on sessions that were dedicated exclusively to pharmacology and sessions where pharmacology was integrated with other disciplines.

We defined pedagogies so that data were represented consistently across the schools. For example, when we discussed Team‐Based Learning (TBL) as a pedagogy, we agreed to only count TBL sessions that mostly follow the trademarked TBL^®^ process with individual readiness assurance tests (iRAT), group readiness assurance tests (gRAT), and application exercises that follow the 4S model.^17^

### 2.3 Application of cognitive engagement framework

**FIGURE 1:**
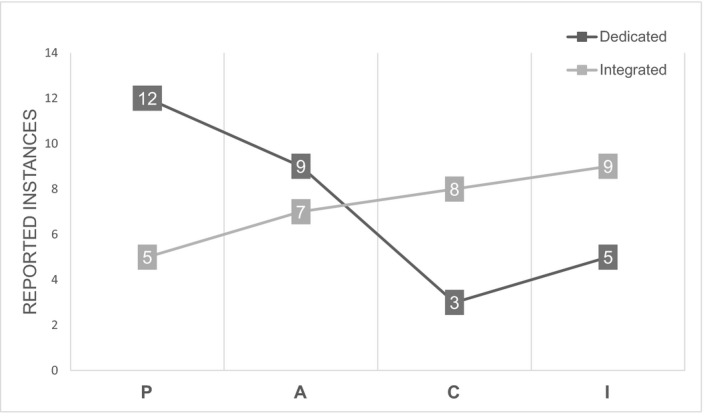
Distribution of reported pharmacology pedagogies according to the ICAP framework (I = Interactive, C = Constructive, A = Active, P = Passive^13^). Categorizing the pedagogical methods reported by the different institutions in this study based on the ICAP framework yields a model for evaluating the level of active and interactive learning used in dedicated and integrated pharmacology sessions

To determine whether levels of cognitive engagement varied between integrated sessions or sessions dedicated to pharmacology, we assigned each pedagogy to a cognitive engagement category of interactive, constructive, active, or passive, based on the ICAP framework.^13^ We calculated a “score” for each category (interactive, constructive, active, or passive) by tallying the number of pedagogies that are used in our curricula for each category (Figure 1 and Table 2). We further parsed the scores by whether the pedagogy was used in integrated pharmacology sessions or sessions dedicated exclusively to pharmacology.

## 3 RESULTS

### 3.1 Identification of key questions for pharmacology curricular designers

To identify the key questions for our descriptive study, we searched for detailed descriptions of incorporation of pharmacology within integrated preclerkship curricula across medical schools. We conducted a survey of the literature looking for works describing entire pharmacology curricula in preclerkship medical programs. There were only two dozen of such works, but many of these papers described approaches to clinical therapeutics or pharmacogenomics rather than basic pharmacology. There were five studies describing detailed curricular pharmacology trends in Australia, the UK, Mexico, and Europe.^18^, ^19^, ^20^, ^21^, ^22^ In reviewing these works, we noted differences among countries in overall curricular representation of pharmacology and clinical therapeutics. We observed that there were few details about the pedagogies used for teaching pharmacology, and even fewer details about assessment of pharmacology knowledge. Finally, we noted that some countries have begun to provide a standardized national drug list. This survey of the literature highlighted several key questions that need to be addressed when considering pharmacology curricular design (Box 1).

BOX 1 **Key questions for pharmacology curricular designers**

● How much pharmacology representation is required in an integrated curriculum, and how is this distributed between sessions dedicated solely to pharmacology versus sessions where pharmacology is integrated with other disciplines?
● What pedagogies are best suited for pharmacology instruction in medical curricula? Does this vary at the session level depending on whether the session is dedicated to pharmacology or integrated with other disciplines?
● How can pharmacology‐specific content be effectively assessed across an integrated curriculum?
● What resources should be provided to students, including during assessments?

### 3.2 Multi‐institutional pharmacology curriculum inventory

Importantly, during our literature survey, we noted that there was no singular, multi‐institutional study describing the pharmacology curricula at allopathic medical programs in the United States. To answer some of these key questions we identified in Box 1, we created a detailed, comparative curriculum inventory (Table 1). This inventory is a multi‐institutional analysis of pharmacology curricula in US medical schools. This inventory was discussed by the authors in monthly collaborative video meetings until consensus was reached that the most important aspects of each pharmacology curriculum were captured, and the key questions from Box 1 were addressed. Specific definitions for the pedagogies described in the inventory are listed separately (Table 2).

**TABLE 1:** Preclerkship curriculum survey of seven pharmacology programs at U.S. Allopathic Medical Schools

Institution	1	2	3	4	5	6	7
Type of curriculum	Integrated Pass/Fail	Integrated Pass/Fail	Integrated Pass/Fail	Integrated Pass/Fail	Integrated Pass/Fail	Integrated Pass/Fail	Integrated Pass/Fail
Length of curriculum	11 months	24 months	17 months	17 months	18 months	18 months	17 months
# Of students per class	☒ 0–99 ☐ 100–199 ☐ 200+	☐ 0–99 ☒ 100–199 ☐ 200+	☐ 0–99 ☒ 100–199 ☐ 200+	☐ 0–99 ☒ 100–199 ☐ 200+	☒ 0–99 ☐ 100–199 ☐ 200+	☒ 0–99 ☐ 100–199 ☐ 200+	☐ 0–99 ☒ 100–199 ☐ 200+
Number of faculty teaching >10 h of pharmacology	1	1	3	2	1	2	3
Focus of faculty appointment for these educators	Education	Education	Education and Research	Education	Education	Education	Education
Total number of sessions dedicated entirely to pharmacology	10	85	82	33	47	76	63
Dedicated pharmacology teaching modalities, ranked by frequency (1=most frequent)	Blended learning Lecture Small Group, Not Facilitated Individual Asynchronous, Not Facilitated Small Group, Facilitated TBL PBL Simulation	Individual Asynchronous, Not Facilitated ABLE Blended learning	Lecture ABLE Simulation Small Group, Not Facilitated	Individual Asynchronous, Not Facilitated ABLE Small Group, Facilitated Lecture	Lecture Individual Asynchronous, Not Facilitated ABLE	Individual Asynchronous, Not Facilitated Lecture ABLE Games	Lecture ABLE Small Group, Facilitated Individual Asynchronous, Not Facilitated
Number of integrated sessions including pharmacology	77	112	14	36	80	27	19
Integrated teaching modalities including pharmacology, ranked by frequency (1=most frequent)	ABLE PBL Lecture Small Group, Not Facilitated Small Group, Facilitated Individual Asynchronous, Not Facilitated Games TBL Simulation	Blended learning ABLE Small Group, Facilitated Simulation TBL	Lecture ABLE Simulation Small Group, Not Facilitated	ABLE Small Group, facilitated	Lecture Individual Asynchronous, Not Facilitated Simulation Small Group, Facilitated Small Group, Not Facilitated	TBL ABLE Simulation	Lecture Small Group, Facilitated
Summative exam questions in non‐organ system science courses	20% (83/420)	25% (30/120)	14% (74/523)	n.a.	8% (17/207)	11% (36/337)	n.a.
Summative exam questions in organ system science courses	14% (34/240)	28% (255/907)	11% (208/1988)	10% (18/175)	10% (69/724)	14% (156/1113)	22% (132/602)
Assessment of discipline competency							
Performance across course exams is tracked	☒	☐	☒	☒	☐	☒	☒
Pharmacology competency required	☒	☐	☒	☐	☐	☐	☒
Pharmacology competency suggested	☐	☐	☐	☒	☐	☐	☐
Formative questions available throughout curriculum	☒	☒	☒	☒	☒	☒	☒
Completion of formative assessments is required	☒	☐	☒	☒	☒	☐	☐
Multiple‐choice questions available throughout curriculum	☐	☒	☒	☒	☒	☒	☒
Other question types available throughout curriculum	☐	☐	☒	☒	☒	☐	☐
Separate pharmacology assessments	☒	☐	☐	☐	☒	☐	☐
CBSE used for progress testing	☒	☐	☒	☒	☒	☒	☐
NBME pharmacology subject testing	☐	☐	☐	☐	☐	☐	☐
USMLE step 1 timing	After clerkships	Preceding clerkships	Preceding clerkships	After clerkships	Preceding clerkships	During clerkships	Preceding clerkships
Pharmacology textbook resources (A = available through library, R = required reading)							
Katzung’s basic & clinical pharmacology	A	A	R	A	R	R	A
Goodman & Gilman’s pharmacologic basis of therapeutics	A	A	A	A	A	A	A
Katzung & Trevor’s pharmacology examination and board review	A	A	A	A	A	A	A
DiPiro’s pharmaco‐therapeutics			A		A		
Access medicine	A	A	A	A	A	A	A
Thieme pharmacology					A	R	
Other resources	Online Med Ed, Sketchy, UWorld, Pharmacology World	UWorld ScholarRx	UWorld	UWorld	Boards & Beyond, UWorld, Firecracker	USMLEasy, ExamMaster, UWorld	UWorld
Students (%) rating pharmacology as ‘good’ or ‘excellent’ preparation for clinical clerkships on AAMC graduation survey (2018–2020 Average, National Average = 78%)	87%	89%	90%	90%	84%	87%	90%

ABLE, Application‐Based Learning Exercises; CBSE, Comprehensive Basic Science Exam; NBME, National Board of Medical Examiners; PBL, Problem‐Based Learning; TBL, Team‐Based Learning.

**TABLE 2:** Definitions of the pedagogical techniques employed at the institutions in this study

Teaching technique/strategy	Definition	Examples	ICAP^a^ designation
Individual asynchronous, not facilitated	Provided or curated by pharmacology educator: video, click‐through powerpoints, podcasts, pre‐reading etc.	Instructor‐created videos or podcasts, reading assignments, third‐party content	P
Lecture	Live lectures provided by pharmacology content experts.	Traditional didactic lectures with or without audience response questions	P
Games	Competition‐based application events.	Jeopardy!^®^, Kahoot^®^	A
Small group, not facilitated	<12 students without faculty facilitator	Patient‐oriented problem‐solving (POPS), self‐directed learning groups	I
Small group, facilitated	<12 students with faculty facilitator	Case‐based learning in small groups with pre‐defined objectives	C
PBL	Small group problem‐based learning sessions following the classic descriptions^33^	Case‐based learning in small groups without pre‐defined objectives	I
TBL	Large group team‐based learning with iRAT/tRAT, application exercises following 4S models^34^	Team‐based learning in large groups	C
Application‐based learning exercises (ABLE)	May be case‐based session or other type of application‐based learning that does not ascribe to PBL/TBL definitions. May or may not have an individual/team quiz component. Large group.	Clinical vignette style questions presented in large group discussion format; TBL application exercise without readiness assignments or assessments	A
Blended learning	Any combination of one individual asynchronous event with one ABLE event.		A
Simulation	Includes use of computer‐controlled mannequin, standardized patients or both.		I

^a^ I = Interactive, C = Constructive, A = Active, P = Passive.^13^

The length of the preclerkship component of the medical school curriculum varies widely among our institutions (ranging from 11 to 24 months), as does the size of our classes (ranging from 50 to 180 students/class). Despite these differences, there are clear areas of concordance. For example, every institution in this study has an integrated curriculum and uses several different active learning teaching methodologies framed within a clinical context to deliver pharmacology content. This is not surprising, given the trend toward increased curricular integration of basic and clinical sciences, and the fact that pharmacology naturally fits into case‐centric active learning exercises. We also discovered many commonalities in pharmacology texts and recommended question banks (Table 1).

Although each school follows an integrated curriculum map, we found that each school also had some pharmacology sessions that were integrated with other disciplines (integrated), and other pharmacology sessions that were dedicated entirely to pharmacology (dedicated). The number of sessions dedicated to pharmacology varied significantly between our institutions (ranging from 10 to 85 sessions with an average of 57 sessions), as did the number of integrated pharmacology sessions (ranging from 14 to 112 sessions with an average of 52 sessions). In total, we have an average of 109 sessions tagged to pharmacology in our preclerkship curricula, fairly evenly split between dedicated and integrated pharmacology sessions.

Another prominent difference among our institutions is how pharmacology content is represented on assessments, and how pharmacology performance on assessments is tracked. The percentage of pharmacology questions on summative exams in organ system courses ranged from 8% to 25%, which is somewhat consistent with the representation of pharmacology in national USMLE Step 1 exams (16%–23%) (https://www.usmle.org/step‐1/#content‐outlines). Our data agrees with previously published work that pharmacology representation on assessments can have a powerful effect on student perceptions and behavior regarding studying and valuing pharmacology.^23^ Tracking pharmacology performance across assessments differs dramatically among our institutions. Among our working group of seven medical schools, 57% track pharmacology performance across foundational science exams longitudinally, but longitudinal pharmacology competency is only required at 29% of our schools and recommended at an additional 14%.

Another interesting finding that represents a large change in pharmacology education, is that over 85% of the faculty authoring this perspective had primary appointments as educators.^24^ This emphasizes the shift that has occurred in institutions, from teaching as part of the responsibilities of a researcher, to one in which the faculty member is not only an expert in the subject but also in pedagogy. The role of these primary educators to function as a liaison between clinical faculty and learners is invaluable in an integrated curriculum; they help to connect clinical faculty educators to sessions relevant to their specific areas of expertise, and they work collaboratively with clinical faculty to write higher‐order assessment questions. This change in the fundamental roles of pharmacology educators is not trivial and may have profound effects on the ability to effectively deliver active learning‐based sessions.^25^ The designation of a foundational biomedical sciences educator comes with the expectation that the faculty member is active in professional development, including conference attendance and scholarly activity.

### 3.3 Integrated sessions have higher levels of cognitive engagement

Integrated curricula contextualize learning in such a way that students are more engaged with foundational science content and learners’ cognitive outcomes are improved, but it is unclear whether this trend persists down to integration at the session level.^5^, ^26^ Because we identified an even split between sessions dedicated entirely to pharmacology (dedicated) and sessions where pharmacology is integrated with other disciplines (integrated), we wanted to determine whether more cognitively engaging pedagogies were used in our integrated pharmacology sessions as compared to our dedicated pharmacology sessions.

To that end, we applied the ICAP Framework to our described pedagogies, assigning each pedagogy to a specific category of cognitive engagement: interactive, constructive, active, and passive (Table 2). Interactive pedagogies involve dialoging, where students “co‐infer” with peers to develop knowledge that neither partner knew previously. Constructive pedagogies are also generating, where new knowledge is created through inferring, comparing and contrasting, and the like, just beyond what was previously encoded. These pedagogies do not require a group setting, and they may not be as highly generating as interactive pedagogies. Active pedagogies involve manipulating information, where existing knowledge is integrated and emphasized. Finally, passive pedagogies are those that involve isolated storing of information.

To adapt our reported pedagogies to the ICAP framework, we tallied the number of times that each specific category of cognitive engagement was represented in the teaching pedagogies listed for dedicated or integrated pharmacology sessions to create a “score” for each category of cognitive engagement. We found that dedicated pharmacology sessions are higher in passive and active pedagogies, where integrated sessions are higher in interactive and constructive pedagogies (Figure 1).

## 4 DISCUSSION

Herein we have described our efforts to identify trends in curricular design of pharmacology content across seven allopathic medical schools in the United States. Based on our individual institutional practices and literature review, four themes became apparent: the value of dedicated pharmacology educators, the effect of integration on the preclerkship curriculum, the heterogeneity of pharmacology assessment, and the debate about resources. Discussion of these themes can help guide both pharmacology education approaches and future research.

### 4.1 Theme 1: *The value of dedicated pharmacology educators*

One of the findings in our analysis was the proportion of faculty (6/7) who had appointments primarily as educators. In addition to being pharmacology content experts, educators are versed in pedagogy, and bring their knowledge of curriculum design, assessment, and teaching techniques to develop effective strategies for teaching.^27^ This is important to note, since studies have shown that dedicated educators have engaged in practices that have been shown to have a measurable effect size on learning, including participation in professional development (*d* = 0.62), development of practices for clear teaching (*d* = 0.75), and building relationships with individual students (*d* = 0.72). Notably, teacher subject matter knowledge had significantly less of an effect (*d* = 0.09) on student learning.^28^

In all of our schools, students rated their perception of pharmacology preparation for clinical clerkships as good to excellent, with percentages well above the national average on the AAMC Graduation Questionnaire (GQ, Table 1). The GQ data presented reflect years from 2018 to 2020 in which the curriculum survey data described are applicable and available. We attribute this data, in large part, to the commitment of our medical schools to employing basic science educators and giving them the protected time required to engage in scholarly teaching.

Since there is an increasing trend to condense preclerkship curriculum time to about 12–18 months, having designated foundational science educators ensures that the pharmacology discipline is not neglected within the curriculum, and that contact time with students is used in the most effective manner based on scholarly approaches to teaching. We have shown, using the ICAP framework, that integrated pharmacology sessions are higher in interactive and constructive pedagogies. While these pedagogies are more cognitively engaging for the learner, they require a higher level of training and faculty development to execute than less engaging pedagogies like a didactic lecture. These professional skills are easily provided by a dedicated pharmacology educator.

An important role of the pharmacology educator is identifying areas where conventional pedagogical tools are not adequate to effectively deliver material in the new curricular reality. There is a need for more comparative curricular research, conducted by pharmacology educators, on pharmacology integration into the preclinical medical curriculum. We anticipate a need for centralized repositories like the AAMC Curricular Inventory (https://www.aamc.org/data‐reports/curriculum‐reports/report/curriculum‐reports) to include data on teaching pedagogies, hours, and assessments at the discipline level, where it is most useful for subject matter experts who are doing the bulk of the pharmacology content creation, design, and teaching.

### 4.2 Theme 2: *The effect of integration on the preclerkship curriculum*

All schools in this study utilize active learning formats to supplement/reduce the amount of didactic classroom time required in the integrated curriculum (Table 1). From our discussions, it became evident that changes in the format of foundational sciences teaching are predominantly driven by changes in accreditation requirements that require reduced contact time with learners and more active learning methodologies. The shift to integrated curricula has required both horizontal integration of pharmacology with other foundational sciences, and vertical integration of pharmacotherapeutics into clinical sciences during the clerkship training years.

Reducing time for individual biomedical science disciplines has both pros and cons. It favors collaboration and promotes integration out of necessity. The creation of cases in collaboration with clinicians ensures that relevant knowledge is presented; however, integrated cases can also reduce the role of pharmacology down to a simple drug choice without providing a thorough rationale for drug selection, potential adverse drug events, and discussion of appropriate alternative therapies. In theory, this achieves the goal of curricular integration (i.e., the development of knowledge that is relevant and meaningful to clinical practice); however basic pharmacodynamic and pharmacokinetic principles are often not revisited during the clerkship years. It would be ideal if the horizontal integration that occurs by integrating basic pharmacology in the preclerkship curriculum would extend with stronger vertical integration of clinical pharmacology in the clerkship curriculum.

Time reduction in curriculum integration also forces pharmacology educators to make difficult decisions about selecting only minimal content beyond that which is deemed essential on USMLE Step 1, at the risk of losing the very structure of the discipline. In addition, they must contend with a continuously shifting landscape of drug approvals and withdrawals, student stress over cognitive overload, and the ever‐increasing amount of drug names and classes. Presently, there is not a national consensus on how to make these curricular decisions. While there are existing pharmacology knowledge objectives with suggestions of drugs to cover from the Association of Medical School Pharmacology Chairs, the time constraints of integrated curricula seldom allow for coverage of drugs beyond the most frequently encountered and prototypical ones. Thus, pharmacology educators are left making isolated choices regarding drugs to include for instruction and assessment. National standards for pharmacology education in an integrated curriculum, like those proposed for pathology, would be tremendously useful to both educators and learners.^29^

### 4.3 Theme 3: *Heterogeneity of pharmacology assessment*

Our monthly discussions revealed that we used some common forms of summative assessments such as the NBME Comprehensive Basic Science Exam (CBSE) as well as USMLE Step 1 data to assess pharmacology‐specific performance. All of our institutions had a pass/fail grading system for preclerkship exams and the percentage of pharmacology questions in foundational basic science courses ranged from 0% to 25% (Table 1), while those in organ‐systems courses ranged from 8% to 25%. One of the topics that frequently arose in our discussions regarding assessments was whether students should be provided resources like drug lists during exams so that they focus less on memorization and more on higher order thinking according to Bloom's taxonomy (e.g., application and analysis). A counter argument is that there are certain drugs that clinical providers are expected to recall, and it is important for students have this knowledge base prior to entering the clerkship curriculum. Unfortunately, we could not arrive at a consensus on this topic, reflective of the larger debate on this issue and indicating the need for more discussion from pharmacology educators at the international level.

Another issue impacting assessment is the increasing use of non‐curricular third‐party resources, which students often use to direct and focus their study for assessment.^30^ As seen in Table 1, most schools provide students with access to electronic texts via subscription services. The non‐curricular third‐party resources that students frequently use are either purchased by the school or by individual students using discounts often provided to the school. It causes student angst when there are discrepancies between internal assessments and non‐curricular third‐party resources. One avenue for intervention is to be more deliberate and effective in validating internal assessments, correlating them with performance on national board exams, and keeping students informed about these relationships.^6^, ^31^, ^32^, ^33^ We anticipate that this tension will be resolved, in part, due to the impending changes in both the USMLE Step 1 and COMLEX grading to pass/fail.

### 4.4 Theme 4: *Debate about resources*

A recent publication predicted that all students will use a common online curriculum as we reimagine medical education.^34^ We do see a trend where students in our programs use very similar resources (e.g., First Aid, USMLE‐Rx, Sketchy, Pathoma, UWorld) in preparation for national board exams, regardless of whether the school provides these or not. While these are valuable resources for board review, we would like to point out that some of these are discipline‐specific resources lacking the integration and application that is the hallmark of enhanced cognitive engagement according to the ICAP framework. In addition, there is often significant heterogeneity in the quality and focus of these resources. Too often, they focus on simple memorization and they often lack the context that can be provided within a structured curriculum. Therefore, these tools cannot replace the institution‐specific integrated sessions where learners can apply multiple foundational sciences to a patient scenario in the presence of faculty available for questions and elaboration. This focus on application will become even more important in institutional settings given the recent move of the USMLE Step 1 to a pass/fail system.

The extent to which a student's national board exam performance is dependent on outside resources versus the institutions’ own curriculum is unknown. It behooves us to study this in more detail as student utilization of outside resources increases. Also, the COVID‐19 pandemic caused a rapid shift to remote online teaching, and the modalities used to deliver pharmacology may markedly change in the coming years. The corresponding impact of the COVID‐19 pandemic on the utilization of outside resources also remains to be elucidated. Thus, the impact and student perception regarding outside resources versus institutional curriculum on student performance must be further investigated in the years ahead.

## 5 CONCLUSIONS

Pharmacology, like many other foundational science disciplines, is increasingly being taught not as a stand‐alone course, but rather as an integrated thread, focused on several core competencies.^29^ Our data suggest that integration at the session level with multiple foundational science disciplines can enhance cognitive engagement. Thus, this approach has many merits, however, we believe it also has the distinct disadvantage of fragmenting and diluting the flow and structure of topics in pharmacology. Having a better understanding of how to deliver topics most effectively in pharmacology to harmonize with other foundational science disciplines and retain a coherent structure and message is vitally important. Multi‐institutional, data‐driven analysis of teaching techniques best suited for specific pharmacology topics is needed. It is our hope that development of such evidence‐based practices or guidelines could help pharmacology educators world‐wide to improve students’ knowledge acquisition, retention, and application in the clinical setting.

Through our survey of the literature, and by comparing curricula at our individual institutions, we have taken the initial step of identifying emerging themes facing pharmacologists in the changing medical school environment. While our institutions utilize different approaches to teaching and assessing pharmacology, student ratings of pharmacology preparation in the GQ are overall very positive, indicating that one size does not fit all, and that institutional control of pharmacology educational materials permits maximal flexibility and integration for local learners according to the unique culture of each institution.

## ETHICAL REVIEW

Although no human subject data were used in creating this perspective, institutional approval and IRB review, when requested, was conducted at each institution.

## CONFLICT OF INTEREST

The authors have no conflicts of interest but have made the following disclosures in the manuscript.

## DISCLOSURE

MWL is the owner and creator of Pharmacology World™ Videos LLC and the creator of the Integrated Pharmacology Atlas™ medical education tool. KMQ and JBB are consultants with ScholarRx. NZ is an item writer/reviewer for the NBOME (National Board of Osteopathic Medical Examiners).

## Data Availability

Data sharing is not applicable to this article as all data created or analyzed in this study are detailed in the tables in the manuscript.
